# Comorbid opioid use is undertreated among forensic patients with schizophrenia

**DOI:** 10.1186/s13011-018-0177-y

**Published:** 2018-11-06

**Authors:** Kristiina Kivimies, Eila Repo-Tiihonen, Hannu Kautiainen, Jari Tiihonen

**Affiliations:** 1 Department of Forensic Psychiatry, University of Eastern Finland, Niuvanniemi Hospital, Niuvankuja 65, FI-70240 Kuopio, Finland; 2 Department of General Practice, Unit of Primary Health Care, Helsinki University Central Hospital, University of Helsinki, PO Box 20, Helsingin yliopisto, FI-00014 Helsinki, Finland; 3 Department of Clinical Neuroscience, Karolinska Institutet, Byggnad R5, SE-171 76 Stockholm, Sweden

**Keywords:** Opioid replacement treatment, Schizophrenia, Forensic psychiatry

## Abstract

**Background:**

Substance use disorders are associated with poorer clinical outcomes in patients with schizophrenia. There is no specific treatment for amphetamine or cannabis use disorder, but methadone and buprenorphine are used as replacement therapy in the treatment of opioid dependence. Our aim was to study whether patients with schizophrenia have received opioid replacement therapy for their opioid use disorder.

**Methods:**

The study sample consisted of 148 individuals diagnosed with schizophrenia who were in involuntary psychiatric treatment as forensic patients in Finland in 2012. The proportion of the study sample with comorbid opioid use disorder having received opioid replacement therapy prior to their forensic psychiatric treatment was compared to the available information of opioid dependent patients in general. The data were collected from forensic examination statements, patient files and other medical registers retrospectively.

**Results:**

Of the study sample, 15.6% (23/148) had a history of opioid use disorder, of whom 8.7% (2/23) had received opioid replacement treatment (95% confidence interval (Cl): 1.1–28.0), even though opioid use disorder had been diagnosed in the treatment system. According the available information the corresponding proportion among patients with opioid use disorder and using substance use disorder services was 30.4% (565/1860, 95% Cl: 28.3–32.5). The fraction of patients receiving opioid replacement therapy was significantly lower among patients with schizophrenia (*p* = 0.022).

**Conclusions:**

Opioid replacement therapy was seldom used among schizophrenia patients who were later ordered to involuntary forensic psychiatric treatment. More attention should be paid to the possible use of opioids when planning treatment for patients with schizophrenia.

**Trial registration:**

Our study is not a randomized controlled trial (but a register-based study); thus the trial registration is not applicable.

## Background

Schizophrenia is a severe mental disorder, which affects the individual’s way of thinking, feeling and behaving in many ways. It is one of the leading causes of years lived with disability [1]. About half of the patients with schizophrenia have problems with substance use [2–4].The prevalence rates for the comorbidity vary between countries. In general, the prevalences are found to be lower in Europe than in the United States [5]. These comorbidities are associated not only with poorer clinical outcomes but also with the increased morbidity and mortality. Patients with comorbid substance use disorder are at a higher risk for medication noncompliance, they have more re-hospitalizations, they have poorer quality of life, and the rate of suicidality and violent behavior is higher [4–12].

Most of the studies concerning comorbid substance use disorder among patients with schizophrenia are focused on the assumed association between cannabis use and the development of schizophrenia, and less attention has been paid to other specific substances, although opioids are the most prevalent drugs among drug users in the European Union [13]. Also, when estimating the burden of disease consequent of drug dependence, the highest estimated global burden was attributable to opioid dependence [14]. Opioid users have a 15-fold increased risk of all-cause mortality when compared to the general population [15], and the risk is still high if the patient is on a waiting list for replacement treatment, indicating an urgent need of the therapy [16]. Detoxification is not an adequate treatment of substance dependence on its own, since relapse is almost inevitable. Opioid replacement therapy, combined with psychosocial assistance, has been found to be the most effective of the treatment options available [17]. World Health Organization (WHO) has stated that pharmacological treatment of opioid dependence should be accessible to all those in need, including those incarcerated and in other closed institutions. Also, according to National Institute for Health and Care Excellence [18], comorbid mental health problems should be treated alongside opioid dependence.

No specific treatment for amphetamine or cannabis use disorder has been demonstrated, but buprenorphine and methadone are used as a replacement therapy in the treatment of opioid dependence. Research has shown the effectiveness of buprenorphine and methadone replacement therapy in reducing mortality and criminal activity, improving physical and mental health, and in decreasing illicit drug activity [19–27], but there are only few studies on opioid replacement therapy among schizophrenia patients with comorbid opioid use disorder. Also, there are no previous studies on the prevalence of this treatment among persons with schizophrenia compared to other persons with opioid use disorder.

The combination of methadone and clozapine was successful when five schizophrenia patients with opioid addiction were treated [28]. Also, according to the study of Gerra et al. [29], the rate of heroin-addicted patients with schizophrenia who remained in replacement treatment and who obtained early full abstinence was higher when olanzapine was used compared to those who were treated with haloperidol. It has been stated that treatment of comorbid opioid use disorder in patients with schizophrenia should be a combination of case management and medical therapy [30]. In a study of adherence to methadone maintenance treatment, persons with a severe mental disorder as schizophrenia have significantly higher adherence to treatment, maybe because they are more closely monitored by health or social care professionals when compared to those without this comorbidity [31].

Our aim was to investigate the prevalence of opioid replacement therapy obtained by patients with schizophrenia and comorbid opioid use disorder prior to their forensic psychiatric treatment, and in other words, to find out whether this patient group had received similar pharmacological treatment for their opioid use disorder as compared to other patients using substance use disorder services.

## Methods

The permission for the study was granted by the Board of Niuvanniemi Hospital and by the Finnish National Institute for Health and Welfare. The study sample included an annual cohort of patients with schizophrenia spectrum disorder who were in involuntary treatment in Niuvanniemi Hospital needing high-security hospital placement as forensic patients at any time in 2012. Niuvanniemi Hospital is a state mental hospital which is in charge of forensic psychiatric services and forensic mental examinations for the needs of the whole of Finland (www.niuva.fi). Opioid use disorder was defined as harmful use of opioids or opioid dependence. In Finland, the official diagnostic classification is ICD-10, according to which all of the diagnoses were made. In ICD-10, harmful use is described as a pattern of a psychoactive substance use that is causing physical or mental damage to health, and dependence as a cluster of behavioral cognitive, and physiological phenomena that develop after repeated substance use and that typically include a strong desire to take the drug, difficulties in controlling its use, persisting in its use despite harmful consequences, a higher priority given to drug use than to other activities and obligations, increased tolerance, and sometimes a physical withdrawal state [32]. All data concerning the study sample was collected retrospectively from forensic examination statements and patient files by the same person (K.K.), and it was checked if the criteria for harmful used or dependence were fulfilled.

In international comparison the Finnish practice of mental examinations can be considered to be very thorough. Preliminary information concerning persons undergoing mental examination is obtained from the persons themselves and, health care units in charge of previous treatment, and also, with their consent, from their family members, friends, employers etc. (www.niuva.fi). A comparison group to contrast the study sample with was from the available information, according to which 565 of the 1860 persons (30.4%) using substance use disorder services in 2011 were receiving replacement therapy. In this group, the mean age was 33, and the median age 32 years [33]. No individual level data such as age was available from the individuals in the national database of substance use disorder patients. Statistical analysis was conducted using the Fisher exact test. It was considered appropriate since the sample sizes were small.

## Results

The cohort of forensic patients consisted of 148 persons, of whom 95.3% (141/148) were male, 56.1% (83/148) had paranoid, 20.3% (30/148) undifferentiated, 3.4% (5/184) hebephrenic, and 4.1% (6/184) some other type of schizophrenia. A total of 24/184 (16.2%) had a schizoaffective disorder (Table 1). The mean age of the sample was 42.8 years, and the mean age at the time of offense was 30.9 years. The average time from the offense leading to mental examination was about four years.

**Table 1:** Description of data demographics and main diagnoses

	Sample	%
Male	141/148	95.3
Female	7/148	4.7
Type of schizophrenia		
Paranoid	83/148	56.1
Undifferentiated	30/148	20.3
Hebephrenic	5/148	3.4
Other	6/148	4.1
Schizoaffective disorder	24/148	16.2

Of the study sample, 15.6% (23/148) had a history of opioid use disorder documented in health care records, of whom and 8.7% (2/23) had received opioid replacement treatment (95% Cl:1.1–28.0).The corresponding proportion among patients with opioid use disorder using substance use disorder services was 30.4% (565/1860), (95%Cl: 28.3–32.5) (Table 2). The fraction of patients receiving replacement treatment was significantly lower among the cohort of forensic patients (*p* = 0.022).

**Table 2:** Prevalences of opioid replacement treatment

	Fractions of patients receiving replacement treatment	95% confidence interval
Cohort of forensic patients	2/23	1.1–28.0
Patients using substance use disorder treatment services	565/1860	28.3.–32.5

## Discussion

The present study evaluates the prevalence of opioid replacement therapy among patients with schizophrenia and comorbid opioid use disorder. Many studies have focused to the nature of the relationship between cannabis use and schizophrenia but less attention has been paid to other specific substances. The burden of disease consequent of drug dependence is highest when it is question of opioid use disorder [34], and patients diagnosed with this comorbidity belong to the costliest group of all patients with opioid use disorder [35]. Evidence based medications for opioid dependence have been shown to be helpful in patients with severe mental disorder [36], but on the other hand, the retention rate in opioid replacement therapy among patients with schizophrenia at 12 months was less than 10%, and over half of the patients had illicit opioid use during the treatment according to the study of Gerra et al., 2006 [37]. Patients with this comorbidity are more likely to have been homeless and to have had a recent psychiatric hospitalization when compared to other patients [38]. In an Iranian descriptive study of 100 patients with schizophrenia, as much as half of the patients had opioid dependence disorder based on DSM-IV criteria. This high prevalence is probably influenced by the easy access and low price of opioids in Iran. However, it was speculated that patients with schizophrenia may use opioids as self-medication to reduce positive symptoms [39]. The antipsychotic effectiveness of opioid agonists is highlighted in a recent review, in which it is suggested that the use of opioid agonists deserves reconsideration because of their anticraving capability and effectiveness on the psychopathological level [40]. It has also been stated that specific groups, including patients with psychiatric comorbidities, may benefit from optimized and higher doses of buprenorphine to ensure sufficient dosing [41]. It is relevant to combine antipsychotic and opioid agonist medications with psychosocial support [38], since group counselling, contingency management and long-term residential treatment have been shown to have positive effects [42].

In our study, only two of the 23 patients with schizophrenia and comorbid opioid use had received opioid replacement treatment prior to hospital treatment as forensic patients. In comparison, the estimated proportion of persons with opioid use disorder undergoing replacement therapy suggests a treatment coverage rate of about 50% in the European Union [43]. In Finland, opioid replacement treatment is typically initiated in inpatient units, after which the clients are transferred to social outpatient services or health centers. General practitioners and pharmacies are also participating in the treatment process. There is no other monitoring system or register of clients in treatment but data collection on a voluntary and anonymous basis by the centers for prevention and treatment of addiction. Also, the coverage of replacement therapy in Finland may vary between different regions. The proportion of opioid users using substance abuse treatment services and receiving replacement therapy in 2011 was 30.4% of problem opioid users using those services and the mean age of those opioid users was 33.0 years [33]. Information did not cover all substance abuse treatment units. It is not known if the patients in the study sample or in the comparison group received other types of treatments. However, when the effect of adding psychosocial treatment interventions to standard opioid agonist treatment programs was compared, it was found that psychosocial treatment did not offer additional benefits in terms of retaining individuals in treatment, supporting abstinence, or preventing relapse [44].

Mental health care and substance use disorder services are often separated from each other, although the coordination of different services is essential for successful treatment of patients with dual diagnoses. In Finland, substance use services are mostly provided by social welfare. Integrated treatment might have benefits for reducing the risk of violent behavior in these patients, since additive genetic influences are shared between schizophrenia, substance use disorder, and violent behavior. Substance use disorder was defined as either harmful use or dependence since in practice, harmful substance use can be quite severe and sometimes it is challenging to distinguish the two.

The information of persons with opioid use disorder and their treatment is incomplete. In this study, it was presumed that the information available was representative enough since information from different substance use disorder services was not described to be missing from systematic reasons.

Our study cohort is not a complete cohort of all offender patients with schizophrenia in Finland. However, it is well representative of patients requiring high-security treatment, since vast majority of those patients are treated in Niuvanniemi Hospital. Those who do not require high-security treatment are located in medium-security units of communities. According to our study, at least in this subgroup of patients with schizophrenia, opioid agonists were rarely used. The possible use of psychosocial methods in treating opioid use disorder was not studied. The latest estimate of individuals in Finland with problem opioid use is from the year 2012 being 13,000–15,000 [45]. In Finland, the number of patients in opioid replacement treatment has increased being about 30% higher in 2015 than in 2011 [46]. However, recent prevalence rates of opioid users indicate increase at least in heroin use [47], and so it can be expected that the number of opioid users and opioid use disorders is going to grow. However, the results of this study should be interpreted with care, since the study sample is from the year 2012.

There are only a limited number of studies exploring the relationship between opioids and psychoses, and the causal connection between opioid use and psychosis is still unclear. In general, the prevalence of psychoses and comorbid opioid use has been found to be low [48]. In the study of Dalmau and colleagues [49], the prevalence of psychotic illness was high among those with amphetamine and cannabis use disorder but lower among those with opioid use disorder. The lifetime rate of opioid use among persons with schizophrenia was about 12% according to Barnes et al. [2]. Comorbid psychotic illness and opioid use is associated with increased mortality [50].

In guidelines for the pharmacological treatment of opioid dependence [15, 51–53], schizophrenia is not mentioned to be a contraindication for opioid replacement therapy.

## Conclusions

Our results indicate that opioid use disorder is not, at least pharmacologically, treated in the same way among patients with schizophrenia as other persons with opioid use disorder. More attention should be paid to possible opioid use when planning treatments for patients with schizophrenia, since this patient group needs to have effective treatment for their co-occurring mental and substance use disorders to reduce morbidity, mortality, and risky behavior. Further studies of the possible advantages and disadvantages of opioid replacement therapy in patients with schizophrenia and comorbid opioid use are needed.

## Abbreviations

CI: Confidence interval
DSM-V: Diagnostic and statistical manual of mental disorders, fifth version
WHO: World Health Organization
